# Superficial Thrombosis of Pelvic Congestion Syndrome Mimicking Pelvic Abscess

**DOI:** 10.5811/cpcem.2019.4.42527

**Published:** 2019-05-29

**Authors:** Adrian Romero, Janae Hohbein, Shana E.N. Ross

**Affiliations:** * University of Illinois at Chicago, Department of Emergency Medicine, Chicago, Illinois; † Newark Beth Israel Medical Center, Department of Emergency Medicine, Newark, New Jersey; ‡ University of Illinois Hospital & Health Science Systems, Department of Emergency Medicine, Chicago, Illinois

## Abstract

Pelvic congestion syndrome (PCS) is an uncommon illness that is typically diagnosed after chronic pelvic pain. We present a case of superficial thrombosis of pelvic veins from PCS that presented to the emergency department (ED) as a previous diagnosis of pelvic abscess with cellulitis. PCS was diagnosed in the ED by computed tomography after an abnormal point-of-care ultrasound. Here we describe this unusual presentation and our approach to the diagnosis.

## INTRODUCTION

Pelvic congestion syndrome (PCS) has been estimated to account for 33% of chronic pelvic pain.^1^ The diagnosis of PCS is typically defined as chronic pelvic pain lasting greater than six months with visible congestion of pelvic veins.^2^ PCS usually affects multiparous women in the reproductive age group. There have been no reported cases in menopausal women, which may be related to the decline in estrogen that leads to venous dilation.^3^

While the pathology is unclear, multiple cases of PCS have been described in patients with gross dilatation, incompetence, and reflux of the ovarian veins. Pregnancy can be associated with up to a 50% increase in venous capacity, resulting in venous incompetence. 4 Thus, it stands to reason that PCS is related to these venous changes, supporting the potential link between multiparity and PCS. However, as all women with venous congestion do not have pelvic pain, a causal relationship has not been established.

## CASE REPORT

A 52-year-old woman with a history of chronic deep vein thrombosis (DVT) on warfarin, presented to the emergency department (ED) for expedited incision and drainage (I&D) of a mons pubis abscess. She complained of progressively worsening mons pubis pain, redness, and swelling for the prior week. The patient had been seen by her primary care physician at the onset of symptoms and diagnosed with a mons pubis abscess. She was prescribed oral antibiotics (sulfamethoxazole/trimethoprim) and scheduled for an I&D later that day with a general surgeon. However, due to intractable pain after sexual intercourse the previous night, she presented to the ED to have it drained sooner. She denied any systemic symptoms including fevers, chills, nausea, vomiting, dysuria, hematuria, vaginal bleeding, or unusual vaginal discharge. In addition, the patient reported intermittent lower abdominal pain for years, which had not recently changed. On further questioning she admitted to not being compliant with warfarin on multiple episodes in the past, but was compliant at the time of presentation.

Physical exam revealed a middle-aged obese female in no acute distress. Vital signs on presentation were significant for a temperature of 36.1 degrees Celsius, pulse rate of 66 beats per minute, and a blood pressure of 166/82 millimeters of mercury. Evaluation of the genitalia revealed a warm, indurated, fluctuant, 5 centimeter (cm) × 5cm × 4 cm left mons pubis area of swelling with moderate tenderness to palpation, and overlying induration. Point-of-care ultrasound (POCUS) revealed multiple fluid-filled tubular structures tracking into the abdomen and left femoral vein with intermittent areas of color flow.

**Image 1:**
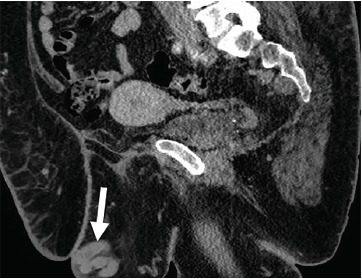
Sagittal cross-section of computed tomography of abdomen and pelvis with intravenous contrast demonstrating dilated pelvic veins (arrow) near the mons pubis.

**Image 2:**
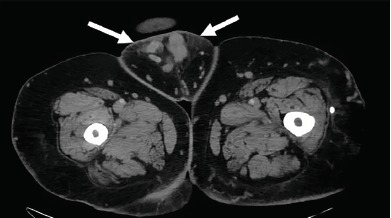
Axial view of computed tomography of abdomen and pelvis with intravenous contrast demonstrating fat stranding (arrows) caused by inflammation from thrombophlebitis.

Given the unusual ultrasound appearance, a computed tomography (CT) of the abdomen and pelvis with intravenous contrast was ordered along with laboratory studies. White blood cell count and international normalized ratio were 8.8×10(3)/microliters and 2.9 respectively. CT revealed multiple prominent collateral venous vessels over the mons pubis and lower abdominal wall consistent with PCS (Image 1 and 2). I&D was not performed and vascular surgery was consulted. Vascular surgery noted a superficial venous thrombosis within the collection of the mons pubis vessels resulting in a clinical picture similar to a mons pubis abscess.

The patient was provided outpatient follow-up with vascular surgery for monitoring and potential thrombectomy. She was also counseled on medication compliance. At follow-up, the pain and swelling had resolved with hot compresses and continuation of anticoagulation therapy.

## DISCUSSION

In the ED it can be easy to anchor on a diagnosis made by an outpatient provider and continue the treatment without being critical of the proposed plan. This patient presented to the ED with a diagnosis and a treatment plan already in motion. A POCUS and thorough history were enough to raise our suspicion and we ordered further studies. This practice should be applied to all suspected abscesses but especially those in the pelvic area of female patients.

In venous congestion of the pelvis, two closely related entities may present as chronic pelvic pain: PCS and vulvovaginal varicosities.^5^ While the pathophysiology of these two conditions is not fully understood the end result is vascular insufficiency of the pelvic venous system. The etiology for vulvovaginal varicosities is multifactorial. Some of the factors linked to vulvovaginal varicosities are proximal venous obstruction, lack of valves in the vulvovaginal veins, and a relative hyperestrogenic state during pregnancy.^6^,^7^ These factors lead to venodilation and venous insufficiency seen on vulvovaginal varicosities. Similarly, PCS is associated with dilation and incompetence of the ovarian veins. No specific causative mechanism has been identified; however, factors similar to the ones seen on vulvovaginal varicosities are believed to play a role.^7^

**CPC-EM Capsule**

What do we already know about this clinical entity?

Pelvic congestion syndrome (PCS) is a diagnosis of exclusion for chronic pelvic pain thought to be associated with dilation of venous system.

What makes this presentation of disease reportable?

Inflammatory changes may confuse the provider into treating as an infectious etiology rather than a purely vascular one.

What is the major learning point?

The use of point-of-care ultrasound can help correctly guide care in cases such as this one where the differential includes such different etiologies.

How might this improve emergency medicine practice?

This case presentation may spur providers to think of PCS as an alternative to pelvic abscess.

Although there could have been an infectious component to our patient’s presentation, as would be the case with cellulitis, abscess, or suppurative thrombophlebitis, the etiology was primarily vascular. It was important to differentiate these two entities since the management varies. This becomes even more critical if the patient is anticoagulated as our patient was, given her history of chronic DVTs.

## CONCLUSION

This case demonstrates the unusual presentation of PCS with the appearance of mons pubis abscess. While the treatment for PCS is not one normally started in the ED, the patient can be counseled on treatment and directed to the correct specialist once the diagnosis is made. The utility of POCUS in PCS is not yet known, but as in our case it completely changed the diagnostic and therapeutic plan.
